# Association of Ambient Air Pollution with Nasopharyngeal Carcinoma Incidence in Ten Large Chinese Cities, 2006–2013

**DOI:** 10.3390/ijerph17061824

**Published:** 2020-03-11

**Authors:** Tianan Yang, Yexin Liu, Weigang Zhao, Zhenjiao Chen, Jianwei Deng

**Affiliations:** 1 School of Management and Economics, Beijing Institute of Technology, Beijing 100081, Chinaadmyxl@163.com (Y.L.); zwgstd@gmail.com (W.Z.); sharon2009@bit.edu.cn (Z.C.); 2 Sustainable Development Research Institute for Economy and Society of Beijing, Beijing 100081, China; 3 Chair of Sport and Health Management, School of Management, Technical University of Munich, Uptown Munich Campus D, 80992 Munich, Germany; 4 Center for Energy and Environmental Policy Research, Beijing Institute of Technology, Beijing 100081, China; 5 Beijing Key Laboratory of Energy Economics and Environmental Management, Beijing 100081, China

**Keywords:** ambient air pollution, nasopharyngeal carcinoma, incidence

## Abstract

Large cities in China are experiencing severe ambient air pollution. Although China accounts for more than 45% of new cases of nasopharyngeal carcinoma worldwide in 2018, few studies have examined the association between ambient air pollution and the high nasopharyngeal carcinoma (NPC) incidence in China. Thus, we aim to investigate whether exposure to ambient air pollution (including nitrogen dioxide, sulfur dioxide, and PM_10_) would significantly affect NPC incidence in large Chinese cities. We collected panel data of ten Chinese provincial cities about local NPC incidence, air pollution level, meteorology, and city profiles during 2006 to 2013 to construct a two-way fixed-effects model to explore the association between ambient air pollution and NPC incidence, as well as possible regional and gender differences behind the association. We found that NO_2_ had the strongest association with NPC incidence, and the relative risks were 2.2995 (95% CI, 1.2567–4.2075) for males and 1.3010 (95% CI, 0.8212–2.0620) for females, respectively. Under cumulative exposure, it was still NO_2_ that had the strongest association with NPC incidence, with a relative risk of 1.8836 (95% CI, 1.2416–2.8577), compared to 1.0857 (95% CI, 0.9474–1.2450) and 1.0547 (95% CI, 0.8790–1.2663) for SO_2_ and PM_10_, respectively. In addition, males were found more sensitive to ambient air pollution than females. We also found that southern Chinese cities were more sensitive to NO_2_ than northern cities, which might be related to a higher humidity there. Our study reveals that exposure to ambient air pollutants like SO_2_, PM_10_, and particularly NO_2_, is significantly positively associated with NPC incidence in China.

## 1. Introduction

Ambient air pollution is correlated with many respiratory diseases [1,2,3]. According to World Health Organization (WHO) data, in 2016, 91% of the world population were living in places where the WHO air quality guidelines levels were not met, which is responsible for an estimated 4.2 million premature deaths worldwide every year [4]. Evidence suggests that ambient air pollution is an underlying factor of nasopharyngeal carcinoma (NPC) [5]. Specifically, studies have shown that people were more susceptible to NPC after long-term exposure to SO_2_ vapor [6]. When inhaled, SO_2_ quickly dissolves in water and forms ${\mathrm{SO}}_{3}^{-}$ and ${\;\mathrm{SO}}_{4}^{-}$, which can cause a series of respiratory diseases, including NPC [7]. PM_10_ (particles with aerodynamics diameter of 10 μm or less) is another pathogenic factor in many respiratory diseases, such as bronchitis, asthma, lung inflammation, and chronic obstructive pulmonary disease (COPD) [8], and can accelerate replication of respiratory syncytial virus and inhibit proinflammatory cytokines [9], both of which are important factors in nasopharyngeal diseases [10]. In addition, PM_10_ particles accumulate, mainly in the upper respiratory tract, while PM_2.5_ and PM_0.1_ penetrate the lower respiratory tract and alveoli [9]. Thus, PM_10_ may be more likely than PM_2.5_ to cause nasopharyngeal carcinoma. Although numerous studies have investigated the association between nitrogen dioxide and lung cancer [11], few have examined its relation to NPC, and even whether such a link is physiologically plausible. Nitrogen oxides may reduce tumor-necrosis-factor compounds, a key pro-inflammatory factor in NPC development [12], and moreover, nitrite and nitrate are formed when nitrogen dioxide is inhaled [13], while nitrite in preserved food increases NPC risk [14].

Although previous studies have linked air pollution to NPC pathogenesis, pertinent empirical studies are rare. A number of questions require empirical evidence. First, is air pollution (NO_2_, SO_2_, PM_10_) a risk factor of NPC [15]? Second, are there any gender differences in the health effects of ambient air pollution [16,17,18], as NPC incidence in men is at least two times that in women [19]? Third, what are the long-term effects of ambient air pollution on NPC incidence [11,20]?

On December 19 2019, we searched on PubMed using terms “air pollution” and “nasopharyngeal” in the title, only three studies are retrieved, two on the harmful effects of air pollution on nasopharyngeal tissue, and only one study, in Taiwan, China, on the association between ambient air pollution and NPC incidence [15]. Then, we searched PubMed using terms “air pollution” and “nasopharyngeal carcinoma” in all fields, and a total of nineteen studies were available, however, most were concerning the relationship between indoor air pollution and NPC.

To date, no study has empirically investigated the health effects of ambient air pollution on NPC incidence in the Chinese mainland. Thus, additional empirical studies, particularly of the Chinese mainland, are urgently warranted. As early as 2015, in the three-year action plan on cancer prevention and control, the Chinese Ministry of Health, included NPC as one of the most serious eight cancers in China [21]. The World Health Organization reported that, in 2018, 129,079 cases of NPC were diagnosed globally, among which 109,221 were in Asia, while 60,558 cases were recorded in China alone, far more than in all other Asian countries combined [22]. Within China, among malignant tumors, NPC incidence and mortality ranked 18th and 17th in 2018 [23], and its five-year survival rate was slightly higher than 50% [19]. Although ambient air pollution may lead to NPC, without relevant empirical research, it is difficult to clarify the association between the two and to develop appropriate measures to reduce the high NPC incidence, and an important pathogenetic factor, such as air pollution, may be unfortunately neglected in controlling NPC incidence, which may result in an inefficient allocation of public resources in nasopharyngeal carcinoma prevention work, and moreover, citizens may be unaware of or underestimate the detrimental effects of air pollution on developing NPC.

Therefore, the present study aims to investigate: (1) The association between the change in ambient air pollution and the change in NPC incidence; (2) gender differences in NPC incidence and its relation to ambient air pollution; (3) the long-term health effects of ambient air pollution; (4) the adjusted relative risk (RR) of NPC incidence in relation to ambient air pollution; and (5) the association between ambient air pollution and NPC incidence growth rate, as a rapid growth of NPC incidence is observed in China. To date, this is the first empirical study of the association between ambient air pollution and NPC incidence in Chinese mainland. 

Most previous studies used a case-control design or cohort analysis to identify, and evaluate potential risk factors for NPC. As the association between outdoor air pollutants and NPC incidence may take some time to become apparent [20], large samples and long-term observations are preferable. Thus, a two-way fixed-effects model, based on the panel data from ten large Chinese cities from 2006 to 2013, is applied in this study. Moreover, a macroscopic city-level model also enables us to control for the effects of local meteorological conditions, like precipitation and humidity, which have proven to be important driving forces of the development of local ambient air pollution levels: For instance, a spread of Chinese air pollution to downwind surrounding regions though air outflow has been found in previous research [24,25,26,27,28].

The rest of paper is arranged as follows: Section 2 presents the methods, Section 3 exhibits the results, Section 4 discusses the results, and Section 5 draws the conclusions.

## 2. Materials and Methods 

### 2.1. Study Area

**Figure 1:**
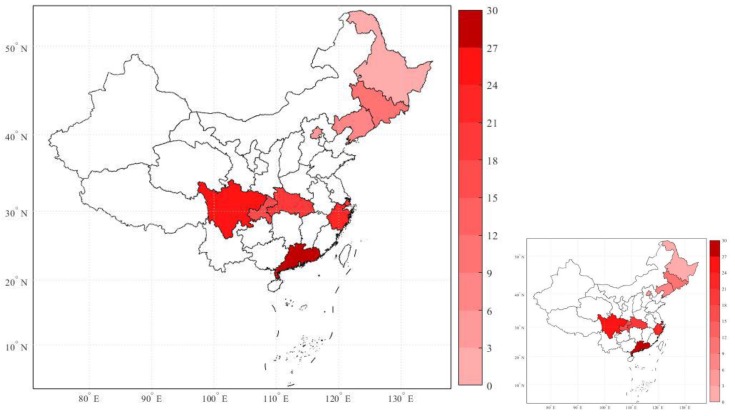
Geographical distribution of the annual average nasopharyngeal carcinoma (NPC) incidence of the ten large Chinese cities, from the period 2006 to 2013. Note: The color-bar on the right represents different degrees of NPC incidence—the deeper the red color, the higher the incidence.

To study the association between ambient air pollution and NPC incidence, ten large Chinese cities (provincial capitals) were selected for collection of data in terms of air pollution, NPC incidence, meteorological conditions, and city profiles from the period 2006 to 2013. The cities are respectively Beijing, Shenyang, Changchun, Harbin, Shanghai, Chengdu, Chongqing, Wuhan, Hangzhou, and Guangzhou, covering a total population of more than one hundred million people. In Figure 1, the geographical distribution of annual average NPC incidence from 2006 to 2013 across ten selected Chinese cities is displayed. 

The cities geographically distributed from southern to northern China as demarcated by the Qinling Mountains-Huaihe River, which indicates that NPC incidence, i.e., the number of cases per 100,000 population per year in a city, was higher in southern cities than in northern cities in this period, as consistent with previous research [19,29].

For the sake of investigating the long-term health effects of ambient air pollution in a period as long as possible, these ten provincial cities were selected conveniently as they are the only ones among all Chinese provincial cities which contain for up to eight years online publicly available data on NPC incidence from 2006 to 2013, as far as we are concerned.

### 2.2. Air Pollution Data

The annual average air pollution concentration was ascertained by using data from fixed air monitoring sites regulated by Chinese Minister of Environmental Protection. The data are published online by Municipal Statistical Bureau of each city, which reports annual average concentrations (μg/m^3^) of various air pollutants, including PM_10_, nitrogen dioxide, and sulfur dioxide. According to the technical guidelines of the Chinese government, these monitoring sites cannot be located near major roads or industrial plants, so that they accurately reflect the general level of urban air pollution. PM_10_, instead of PM_2.5_ and ozone, is selected as an independent variable mainly due to limitation in data accessibility, as the monitoring of PM_2.5_ and ozone is initiated by Chinese government in 2012 at the earliest, before which only data on NO_2_, SO_2_, and PM_10_ are available.

### 2.3. Data on NPC Incidence

Annualized summarized data on NPC incidence were obtained from the annual report of cancer register published by the Tumor Registration and Reporting System, a division affiliated with the Chinese Center for Disease Control and Prevention, which was established by the Chinese government in 1978 to provide real-time data on national incidence and mortality from all-causes. The Tumor Registration and Reporting system was established in 2002 with the approval of the Chinese Ministry of Health and comprises many cancer monitoring sites in numerous cities. If hospitals and health centers at various levels identify new cancer cases or deaths from malignant tumors, reports will be submitted to the management agency of the Tumor Prevention and Reporting System. Until 2022, more than 850 cancer registries will be included in the national cancer register annual report. The NPC incidence, overall and by sex, is reported after age weighting to facilitate comparisons between cities with different age structures. 

### 2.4. Confounder Variables

To control for the effects of meteorology on incidence [24,25,26,27,28], urban meteorological data, including annual hours of sunshine, annual precipitation, and annual average humidity, were obtained from meteorological stations regulated by Municipal Meteorological Office in the ten cities. The monitoring standards are consistent with the international World Meteorological Organization standards. 

Ambient air pollution is aggravated by automobile exhaust gas [11], which may be linked to NPC morbidity. Therefore, we controlled the number of private cars owned per 100 population to control for the effect of urban traffic conditions. Urban green coverage rate and second industrial output were added to control for the confounding effects of urban greenery and urban second industrial development [30]. Tobacco and alcohol consumption are confirmed to be potential pathogenic factors of NPC [31,32], so we adjusted for tobacco and alcohol consumption per capita (adjusted by the historical CPI (consumer price index) index for tobacco and alcohol). Similarly, per capita disposable income (adjusted to baseline by using CPI index history to eliminate inflation) was added to control for the effect of social economic status [33,34,35,36]. Levels of urban education popularization and public medical resources were controlled for by using educational personnel per capita and number of hospitals and clinics as proxies, as they may affect NPC incidence [18,36]. The Chinese government’s determination to curb urban air pollution likely affects air pollutant concentrations [37], and it is reasonable to assume that the government also deals with air pollutants outside our model, such as ozone, which may also affect NPC incidence. Thus, to avoid confounding, the number of urban industrial exhaust-gas treatment facilities was used as a proxy of governmental attempts to curb air pollution. 

### 2.5. Statistical Analysis

Descriptive statistics were generated using the mean, standard deviation (SD), and quartiles of each variable. 

This study used a two-way fixed-effects model [17], based on the panel data from ten large Chinese cities from 2006 to 2013. This method removes possible confounding factors from the error term that remain constant or change synchronously over time for each city.

The two-way fixed-effects model is a generalization of the classical DID (difference-in-difference) model and is widely used in statistics and econometrics, with the following basic form:

Y_it_ = β_0_ + δ_0_ P_it_ + X_it_ + v_i_ + γ_t_ + ε_it_ (1)

The dependent variable Y_it_ is the NPC incidence in city i in the time point *t.* The key predictor variable P_it_ represents the air pollutants examined: NO_2_, SO_2_, and PM_10_. X_it_ is a set of control variables. v_i_ depicts the unobserved fixed effects of an individual city, which remain constant or grow slowly over time with an effect on NPC incidence, such as social customs, rituals, and dietary habits. γ_t_ depicts time-fixed effects and captures unobserved effects of synchronous time trends on NPC incidence across all cities, such as nationwide policies. ε_it_ is the error term. The cluster robust standard deviation has been used to avoid serial correlation and heteroscedasticity. The data were analyzed using Stata 13.1 software.

To acquire an elasticity-interpretation of the effects between air pollution and NPC incidence, the model was fitted on logarithmic values of the original data of all variables, as follows:

LnY_it_ = β_0_ + δ_0_ LnP_it_ + LnX_it_ + v_i_ + γ_t_ + ε_it_ (2)

To ensure that the time-series of all variables were stationary, both the ADF (Augmented Dickey-Fuller) and LLC (Levin-Lin-Chu) unit root test were conducted before formal estimation. After logarithmic processing, two control variables were processed in first difference to keep stationary, including annual disposable income per capita, and tobacco and alcohol consumption. To simulate and capture the long-term effects of ambient air pollution, totally three years’ lagged terms of air pollutants were added.

First, we investigated the effect of ambient air pollution on NPC incidence in the overall population, and then by sex. Considering the possibility of a regional difference in the health effects of air pollutants [17,38], to investigate the difference in sensitivity to ambient air pollution between the southern and northern cities, we further examined whether the interaction between ambient air pollutants with southern city dummy variable would have a significant effect on NPC incidence.

Second, the effect of air pollution on NPC incidence growth rate, overall and by sex, was investigated by treating the dependent variable in the first-order difference after logarithmic processing. Log-difference treatment can be used to estimate annualized growth rates of original time series and has the additional advantage of smoothing the curve as well as partially eliminating heteroscedasticity [39]. Then, interaction plots were drawn for a clearer view of the regional difference in the effect of ambient air pollution on NPC incidence and its growth rate between southern and northern Chinese cities, with simple slope tests done to test the significance of slopes [40].

Third, we investigated the cumulative health effects of ambient air pollution. If P_it_, P_it−1_, P_it−2_, and P_it−3_ respectively refers to the air pollution in the current, lag1, lag2, and lag3 periods, and if we make δ_1_ equal the sum of β_1_, β_2_, β_3_, and β_4_, which are the coefficients of P_it_, P_it−1_, P_it−2_, and P_it−3,_ then β_1_ would equal δ_1_ minus β_2_, β_3_, and β_4_, and the model could be written as:

LnY_it_ = β_0_ + δ_1_ LnP_it_ + β_2_ (LnP_it−1_ – LnP_it_) + β_3_ (LnP_it−2_ – LnP_it_) + β_4_ (LnP_it−3_ – LnP_it_) + LnX_it_ + v_i_ + γ_t_ + ε_it_ (3)

This transformation has been broadly used in finite distributed lag models [41]. In this model, the coefficient δ_1_ represents the cumulative health effects or long-run propensity of exposure to ambient air pollution, for which the confidence interval (CI) can be calculated as per the standard deviation of δ_1_. The cumulative effect is essentially different from the long-term effect in that the latter assumes that the increment of air pollutant concentrations in one period will not extend to the next period, while the former assumes that the concentration increment extends from the current period to future periods.

Finally, to compare present and past findings, we transformed the model from log-log to log-linear, where air pollutants were treated in their original series but control variables remained in their logarithmic form, to calculate the adjusted relative risk (RR) of NPC incidence associated with an increment of 10 μg/m^3^ in the concentrations of air pollutants, as previously reported [42]. The RR and its confidence interval were calculated as per e^βΔx^, where Δx refers to a 10 μg/m^3^ increment in air pollutant concentrations. The RRs for NPC incidence were analyzed in three different models: A one-pollutant model with zero lag, a one-pollutant model with three lags, and a three-pollutants model with three lags to control for the effects of pollutant numbers.

We conducted a further sensitivity analysis to test whether the effect of air pollution would change greatly after model modifications. Models with zero, one, and two lags of air pollution were established to compare with the basic model involving three lags. Then, models adjusted only for meteorological variables were established to eliminate the effect of multicollinearity on relative risks. The result is displayed in the appendix.

The data that support the findings of this study can be downloaded from four Chinese online data repositories: NPC incidence data are from China Health Database, air pollution data and meteorological data are from China City Database, data of control variables are from China Macro economy Database, all of which can be accessed on Chinese Easy Professional Superior (EPS) platform.

## 3. Results

### 3.1. Data Description

The variables are summarized in Table 1. In brief, the incidence was higher in the south than in the north, and higher in men than in women, consistent with previous discoveries [19,29]. PM_10_ pollution was greater in the north than in the south, while nitrogen dioxide pollution was greater in south than in the north. The annual average air pollution concentrations in the ten large Chinese cities far exceeded the requirement of WHO guideline, which set a upper limit of an annual average of 20 μg/m^3^ for PM_10_, 40 μg/m^3^ for NO_2_, and a daily average of 20 μg/m^3^ for SO_2_ [43]. Precipitation and humidity were greater in the southern than in the northern cities, while the duration of sunshine was shorter in the south, consistent with the climatic features of areas located in different geographical latitudes.

### 3.2. Association of Air Pollution with NPC Incidence and Its Growth Rate

Estimates of the associations between air pollution and NPC incidence (the first to fourth column) and its growth rate (the fifth to eighth column) based on a two-way fixed-effects model, after controlling for a series of possible confounding variables, are presented in Table 2, in which all variables were in logarithmic form to eliminate heteroscedasticity and estimate elasticity.

An increase in current period’s NO_2_ concentration is positively associated with both NPC incidence and its growth rate, overall or by sex, with or without interaction terms, with significant influences at the 1% level, except on the incidence in females (*t* = 0.78). For every 1% increase in NO_2_ concentration in the current period, the overall NPC incidence increases by 2.509% (4.18% and 0.599% for males and females, respectively) and overall NPC incidence growth rate increases by 6.458% (8.509% and 7.769% for males and females, respectively). Similarly, the health effects of SO_2_ in the current period are positively associated with both NPC incidence and its growth rate, but not quite statistically significant like NO_2_. As for PM_10_, it seems that it is most strongly associated with NPC incidence of females, instead of males.

The long-term health effects of air pollution are manifested by the fact that all significant coefficients in the lag2 period are positive in relation to both NPC incidence and incidence growth rate. For example, a 1% increase in the concentration of SO_2_ is associated with an increase in NPC incidence from 1.125% to 1.824%, and an increase in its growth rate from 1.993% to 2.759%; and a 1% increase in the concentrations of PM_10_ is significantly related to a 1.077% and 1.908% increase in overall NPC incidence and its growth rate, respectively.

For males, the air pollutant with the strongest influence in the current and lag3 period is NO_2_, and that in the lag1 and lag2 period is PM_10_. For instance, every 1% increase in NO_2_ concentration in the current period increases NPC incidence of males by 4.18%. While for females, possibly due to a comparatively lower incidence versatility compared to males, as displayed in Table 1, the influences of some lagged terms are not as statistically significant as they are for males. However, from the perspective of NPC incidence growth rate, which amplifies the volatility of incidence and captures more slight variations by calculating log-difference, many terms become significant, and it is still NO_2_ that has the strongest influence in the current and lag3 period; moreover, the air pollutant with the strongest effect in the lag2 period is still PM_10_, like in the case of males. We noticed that PM_10_ in the current period was negatively correlated with NPC incidence, except for females, perhaps because of an underestimation due to the failure to consider use of anti-haze masks among citizens, as well as the tendency for people to stay indoors when air pollution is severe [17]. Moreover, the detrimental effect of PM_10_ on men may require a longer incubation period, as from the lag1 period (*p* = 0.068), lag2 period (*p* = 0.078), to lag3 period (*p* = 0.518), PM_10_ is positively associated with NPC incidence. In sum, the result implies that males are more sensitive to the effect of NO_2_ on NPC incidence than females, as all coefficients related to NO_2_ in the third column (males) are larger than that in the fourth column (females).

In addition, we also noticed that the influences of NO_2_ on NPC incidence in the lag1 period are significantly negative, which appears to suggest that the increase in NO_2_ concentration is responsible for the earlier onset of NPC from the next year to this year. Similarly, both NO_2_ and PM_10_ in the lag1 period are negatively correlated with NPC incidence growth rate, perhaps due to a higher cardinality, and many studies have suggested that the exposure–response relationship will become less steep as air pollution levels increase, which may help explain why higher concentrations of air pollution are related to lower NPC incidence growth rate for NO_2_ and PM_10_ in the lag1 period [38,44].

With respect to control variables, average annual disposable income per capita, industrial exhaust-gas treatment facilities, private-car-ownership per 100 population, number of hospitals and clinics, educational personnel per capita, and precipitation are negatively correlated with NPC incidence, while average annual air humidity, hours of sunshine per year, green coverage rate, total output value of secondary industry, and per capita consumption of tobacco and alcohol are positively correlated with NPC incidence. The estimation result of control variables is shown in Table A1.

To investigate the regional difference, three interaction terms consisting of the current-period air pollution with a southern city dummy variable were added, but none were significant unless the interactive term containing PM_10_, which had the lowest *t*-value, was removed. Then, as compared with northern cities, every 1% increase in current NO_2_ concentration in southern cities is associated with another 2.228% increase in NPC incidence, and another 2.259% increase in NPC incidence growth rate, while northern cities appear to be influenced more by SO_2_.

**Figure 2:**
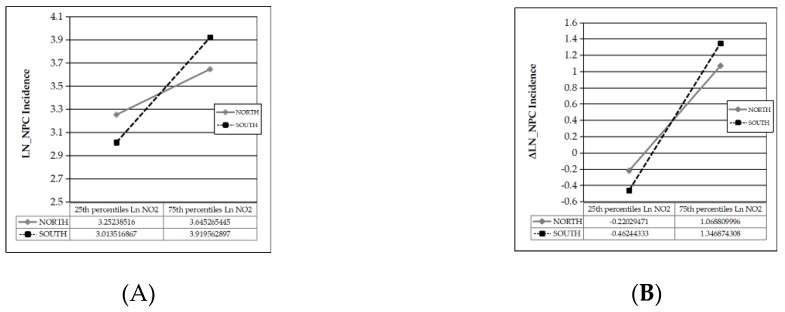
(**A**) Sensitivity of NPC incidence to NO_2_ concentration in southern and northern Chinese cities; (**B**) Sensitivity of NPC incidence growth rate to NO_2_ concentration in southern and northern Chinese cities.

The interactive plots are shown in Figure 2A,B.

In Figure 2A, the sensitivity of NPC incidence to NO_2_ differs between northern (simple slope *t* = 10.922, *p* = 0.000) and southern (simple slope *t* = 11.688, *p* = 0.000) cities. Similarly, in Figure 2B, the sensitivity of NPC incidence growth rate to NO_2_ differs between northern (simple slope *t* = 5.630, *p* = 0.000) and southern (simple slope *t* = 7.345, *p* = 0.000) cities. In the condition that other factors are kept constant at their average, when NO_2_ concentration increases by one interquartile range (IQR) from the average, NPC incidence increases by 7 (≈ e^3.6453^−e^(3.2523 + 3.6453)/2^) per 100,000 population in northern cities and 18 (≈ e^2.7917^−e^(2.7917 + 2.7556)/2^) per 100,000 population in southern cities, indicating a marked regional difference. In the condition that all factors including air pollution level are kept constant at their average, the average NPC incidence is 32 (≈(e^3.6453^ + e^3.2523^)/2) per 100,000 population nationwide, and the average annual NPC incidence growth rate is approximately 42% (≈(1.0688 − 0.2203)/2) nationwide, indicating an increasing trend in NPC incidence over time.

### 3.3. Cumulative Health Effect of Air Pollution on NPC Incidence and its Growth Rate

To better evaluate the long-term cumulative health effects of air pollution, long-run propensity covering four years was calculated for each air pollutant (Table 3). When the concentration of air pollutants increases by 1% annually during the current year and future three years, the cumulative health effect will be a 3.4%, 1.547%, and 2.152% increase in NPC incidence and an 8.899%, 6.315%, and 3.539% increase in NPC incidence growth rate, for NO_2_, SO_2_, and PM_10_, respectively. For overall NPC incidence, the air pollutant with the strongest cumulative health effects is NO_2_, followed by PM_10_ and SO_2_; and for overall incidence growth rate, the air pollutant with the strongest cumulative health effects is also NO_2_, however followed by SO_2_ and PM_10_ successively.

### 3.4. Relative Risks of NPC Incidence in Relation to Ambient Air Pollution

Table 4 summarizes the relative risks of NPC attributable to air pollution, respectively, in a one-pollutant model with zero lag (Model 1), a one-pollutant model with three lags (Model 2), and a three-pollutants model with three lags (Model 3). The cumulative relative risks are shown in Model 4. The four models separately estimate the relative risks of NPC incidence in relation to air pollution, overall and by sex, adjusted for all control variables. In the one-pollutant model with zero lag (Model 1), one-pollutant model with three lags (Model 2), and three-pollutants model with three lags (Model 3), it is NO_2_ that has the largest effect on incidence overall and by sex in the current period, and the health effects are gradually strengthened from Model 1 to Model 3. In Model 3, the adjusted RRs of NPC in relation to NO_2_ in the current period are of 1.5062 (95% CI, 1.2278–1.8478), 2.2995 (95% CI, 1.2567–4.2075), and 1.3010 (95% CI, 0.8212–2.0620), respectively, while SO_2_ is not strongly associated with NPC incidence in the current period and has a significant but weak association with NPC incidence in females (adjusted RR = 1.0001, 95% CI = 1.0000−1.0003) in the lag1 period, and PM_10_ in the current period is most strongly associated with NPC incidence in females, with an RR of 1.2649 (95% CI, 1.0674–1.4990). Taken together, NO_2_ is most strongly associated with NPC incidence either overall, in males, or in females across three models in the current period.

**Figure 3:**
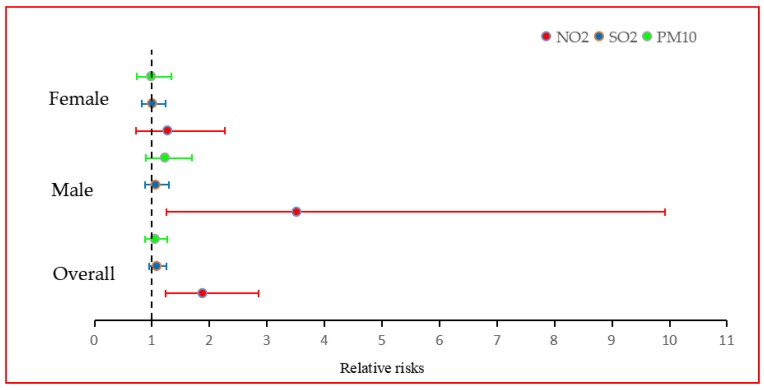
Relative risks of NPC incidence associated with a 10 μg/m^3^ increase in air pollutants concentrations annually lasting for four years.

Model 4 exhibits the cumulative relative risks of NPC incidence overall and by sex, in relation to cumulative exposure to three air pollutants, NO_2_, SO_2_, and PM_10_, specifically with a 10 μg/m^3^ increment in concentration annually lasting for four years. The relative risks of NPC incidence overall associated with cumulative exposure to SO_2_ and PM_10_ are 1.0857 (95% CI, 0.9474–1.2450) and 1.0547 (95% CI, 0.8790–1.2663) respectively, and the cumulative exposure to NO_2_ has a significant positive effect on NPC incidence overall and in males, and a positive but not significant effect in females: the RR values were 1.8836 (95% CI, 1.2416–2.8577), 3.5187 (95% CI, 1.2479–9.9215), and 1.2742 (95% CI, 0.7161–2.2696), respectively. In sum, under cumulative exposure, the air pollutant with the strongest adjusted RRs is NO_2_, both overall and by sex. A forest plot of this result has been drawn in Figure 3.

### 3.5. Sensitivity Analysis

In a sensitivity analysis of models separated by sex with different numbers of lagged terms, the results remain robust, especially those for NO_2_. Exposure to NO_2_ and SO_2_ leads to an increase in NPC incidence in the current period, which is greater in males, while PM_10_ appears more harmful to females (Table A2). Regarding the growth rate of NPC incidence, the effects of air pollutants are largely positive, and the effects of NO_2_ in the current period are all significantly positive, for which the effects are stronger on males than on females (Table A3). In a sensitivity analysis of RRs in models with different numbers of pollutants and adjusted only to meteorological variables, estimates are largely unchanged. In the three-pollutants model with zero lag, the one-pollutant model with zero lag, and the one-pollutant model with three lags, the adjusted RRs for NO_2_ remain quite similar, from 1.1026 (95% CI, 0.9623–1.2634), 1.122 (95% CI, 0.985–1.278), to 1.0862 (95% CI, 0.9831–1.2). In sum, the relative risks associated with NO_2_ are mostly greater than those for SO_2_ and PM_10_, in all but lag1 period (Table A4).

## 4. Discussion

This study is based on a two-way fixed-effects model and panel data from ten large Chinese cities during 2006 to 2013 to examine the association between ambient air pollution and NPC incidence. Several confounding factors, including levels of education popularization, levels of medical treatment, levels of urban greening, consumption of tobacco and alcohol per capita, disposable income per capita, second industrial development, and meteorological conditions are adjusted for. By applying individual fixed-effects and time fixed-effects, we are able to control for most potential confounding factors that cannot be adjusted for in time-series and cross-sectional models, such as percentage of smokers, demographic structure, dietary habits, epidemic and virus status, and nationwide policies [17].

The analysis shows that ambient air pollution, particularly from NO_2_, is strongly associated with NPC incidence in China. For every 1% increase in NO_2_ concentrations, the overall NPC incidence increases by at least 1.706%, and the overall NPC incidence growth rate increases by at least 5.598%. These findings are consistent with previous studies who reported that NO_2_ displayed the strongest association with NPC incidence, or CBD (Cerebrovascular Disease) and CVD (Cardiovascular Disease) mortality, as compared to SO_2_ and PM_10_ [2,15]. The harmful effect of indoor air pollution can support our finding from another perspective: An increased NPC risk has been found associated with daily incense burning (Adjusted OR = 2.49, 95% CI: 1.33–4.66) in women, and the adjusted OR for daily burning with good ventilation (1.35, 95% CI: 0.92–1.98) is much lower than that with poor ventilation (2.08, 95% CI: 1.02–4.24) [45]. Moreover, compared with non-users, frequent wood fuel use is also associated with NPC risk (OR 1.95, 95% CI, 1.65–2.31) [46]. Because only few previous studies examine the relationship between ambient air pollution and NPC, this empirical study enriches the relevant literature and demonstrates new mechanisms underlying NPC pathogenesis [14].

Analysis of sex differences sheds light on the reasons for the marked sex difference in NPC incidence in China and the rest of the world [47,48]. The model estimation results indicate that NO_2_ has a significantly greater effect on men than on women. The NPC incidence and incidence growth rate for males are 6.97 and 1.09 times those of females (Table 2), respectively, perhaps because of physiological differences between sexes [17,18]. Significant gender differences in risk of NPC has also been found in passive smoking in previous research [49]. 

In our analysis of the long-term relationship, the model result shows that NO_2_ in the lag2 and lag3 period, as well as SO_2_ and PM_10_ in the lag1 and lag2 period, have a significant positive association with NPC incidence (Table 2). These findings indicate that exposure to air pollution even three years ago still has significant positive effects on NPC incidence in the current year [20]. As for the cumulative health effect of a 10 μg/m^3^ increase in NO_2_ concentration for the successive four periods, the relative risks for NPC are respectively 3.5187 (95% CI, 1.2479–9.9215) for males, 1.2742 (95% CI, 0.7161–2.2696) for females, and 1.8836 (95% CI, 1.2416–2.8577) overall, higher than that of SO_2_ and PM_10_.

Intriguingly, we are surprised by the finding that humidity, higher in southern cities, is strongly positively associated with NPC incidence and its growth rate (Table A1). This suggests that air humidity may be another important factor affecting the nasopharynx, possibly because in moist air, PM_2.5_ and PM_10_ particles generate more toxic reactive oxygen species through combination with water molecules [50], and so did nitrogen dioxide through similar mechanisms [51]. It is suggested that people should not stay outside for too long on foggy days with heavy air pollution. Future molecular chemistry studies should attempt to explain the role of humidity in NPC incidence. Moreover, by adding an interaction term, we do note that the effect of nitrogen dioxide on NPC incidence is significantly greater in southern cities than in northern cities, which may be explained by the higher humidity in southern cities.

According to the latest nitrogen dioxide map released by European Space Agency on December, 2019, nitrogen dioxide levels over most areas of China, such as the Beijing-Tianjin-Hebei Region, Yangtze River Delta, and Pearl River Delta, was much beyond 100 μmol/m^2^, more serious than that of neighboring countries [52], and highly likely imposing negative health effects on the human body [36]. In densely populated urban areas in China, vehicle exhaust is an important source of NOx, besides industrial waste gas. The current study suggests that NO_2_ may be potential pathogenic factors of NPC: Thus, to ensure public health, regulators must implement restrictive policies to control industrial and automobile exhaust emissions, encourage use of clean energy, and promote popularization of new-energy vehicles to reduce ambient air pollution levels [53,54]. Moreover, the Chinese government should stick to the emission abatement strategy to improve the environmental health [37].

This study has limitations that warrant considerations. First, we only consider the long-term health effects of air pollution across a span of four years. However, future research should use longer delays of air pollution in panel data models to systematically examine the patterns of long-term health effects of ambient air pollution. Second, data of ozone, which is toxic to humans, could not be obtained because of data limitations; however, this does not affect the statistical power of our estimates. Third, although the individual fixed-effects and time fixed-effects capture specific urban characteristics that do not change or change slowly and synchronized changes in all cities over time, they cannot be used to control for factors that change asynchronously across cities, such as the rate of breathing mask use, which may lead to underestimation of the harmful effect of PM_10_ on NPC incidence, but the missing variable would not influence the effect estimation of NO_2_ or SO_2_, as both air pollutants can still penetrate through breathing masks. Fourth, we do not consider the health effects of indoor air pollution. If people prefer to stay at home because of severe ambient air pollution, the health impact of ambient air pollution might be underestimated [17]. Finally, future empirical studies should include more medium-sized cities, to improve the external validity of the conclusions.

## 5. Conclusions

Ambient air pollution is strongly positively associated with NPC incidence in ten large Chinese cities across a long period. The effect of NO_2_ on NPC incidence is stronger in men than in women, which suggests that men are more sensitive to NO_2_. In addition, through as of yet unidentified mechanisms, humidity may increase the harmful effects of air pollution on NPC incidence, which are greater in southern (more humid) cities than in northern cities. Larger panel studies including medium-sized cities and longer periods, and spatial-temporal studies would likely yield additional useful information.

## Figures and Tables

**Table 1:** Summary statistics of variables.

								Northern Cities		Southern Cities		
Variable	Mean	Std	Min	Q25	Q50	Q75	Max	Mean	Std	Mean	Std	F-Stat
★NPC incidence of male(people per 100,000)	5.94	5.92	0.58	2.02	4.99	6.51	25.64	1.90	0.79	8.70	6.31	34.28 ***
★NPC incidence of female	2.40	2.34	0	0.86	1.72	2.87	10.71	0.85	0.37	3.47	2.51	32.00 ***
★PM_10_ (μg/m^3^)	102	18.68	70	89.3	102	111.2	160	109	17.3	97.4	18.28	8.04 ***
★SO_2_ (μg/m^3^)	42.6	13.66	20	31	40.2	52.8	90	42.7	14.28	42.5	13.38	0.002
★NO_2_ (μg/m^3^)	49.7	8.21	30	44	51.1	55.6	70	46.9	9.03	51.5	7.12	6.544 **
★Humidity (share)	68.26	8.03	49	64.25	70	72.75	82	61.28	6.66	73.34	4.23	92.89 ***
★Sunshine (hours/year)	1838.23	541.62	703.8	1487.03	1773.95	2360.48	2699.6	2367.53	185.21	1453.28	356.1	175.8 ***
★Precipitation (mm/year)	1016.87	471.48	318	627.88	998.15	1341.95	2353.6	614.73	151.27	1309.33	404.2	85.53 ***
★Green coverage (share)	37.49	4.09	23.45	35.72	37.96	39.85	47.69	36.81	5.23	37.96	3.1	1.47
★Tobacco and alcohol consumption (yuan)	422.73	152.93	168.05	313.65	392.07	532.2	807.25	378.93	144.96	451.92	152.6	4.57 **
★Private cars ownership (cars per 100)	11.69	10.35	0.67	3.56	8.86	17.4	42.32	10.76	11.38	12.31	9.68	0.43
★Second industry output (billion yuan)	3095	1760	727.5	1762.1	2595.7	3969.1	8027.8	2151.9	1007	3723.9	1878	18.78 ***
★Citizen income (yuan)	44,824	17,540	18,779	31,611	41,900	54,046	93,960	42,681	18,343	46,348	16,990	0.815
★Industrial exhaust gas treatment facilities (a)	2134	1390	444	1057	1864	2598	8917	1617	833	2478	1577	8.03 ***
★Educational personnel per capita	0.02	0.01	0.01	0.02.	0.02	0.02	0.04	0.02	0.01	0.02	0.0043	3.65 *
★Hospital and health-centers (a)	541.54	357.43	221	299	446	633	1502	426.16	138	620.11	433.85	5.962 **

Note: Unit in parenthesis. (**a**): Amount. Std: Standard deviation. Northern cities: Beijing, Shenyang, Changchun, and Harbin. Southern cities: Shanghai, Chengdu, Chongqing, Wuhan, Hangzhou, and Guangzhou. Q25: 25% quantile, Q50: 50% quantile, Q75: 75% quantile. F-stat: F-statistics of one-way ANOVA of variables between southern and northern cities. * *p* < 0.1, ** *p* < 0.05, *** *p* < 0.01.

**Table 2:** Association of air pollution with NPC incidence and its growth rate.

	Ln NPC Incidence				Δ Ln NPC Incidence			
	Overall	Overall#	Male	Female	Overall	Overall#	Male	Female
LnNO_2_	2.509 ***	1.706 ***	4.180 ***	0.599	6.458 ***	5.598 ***	8.509 ***	7.769 ***
	(5.31)	(3.56)	(5.43)	(0.78)	(5.63)	(5.63)	(5.07)	(5.98)
LnSO_2_	0.219	0.504 **	0.345	0.0980	0.0859	0.504	0.0483	0.0835
	(1.13)	(2.67)	(1.28)	(0.34)	(0.30)	(1.28)	(0.11)	(0.68)
LnPM_10_	−0.506	−0.0434	−1.289 *	1.938 **	1.462 *	1.324	1.421	1.831
	(−1.11)	(−0.09)	(−1.86)	(2.58)	(2.22)	(1.75)	(1.46)	(1.69)
LnNO_2__lag1	−0.847 **	−1.109 **	−0.546	−0.779	−1.137 ***	−1.327 ***	−0.856 *	−0.698 *
	(−2.51)	(−2.94)	(−1.36)	(−1.04)	(−5.62)	(−4.41)	(−2.23)	(−1.98)
LnSO_2__lag1	1.416	3.141	−2.002	10.910 *	6.518 *	4.948	8.457	−9.018
	(0.74)	(1.30)	(−0.55)	(2.13)	(1.92)	(1.80)	(1.37)	(−1.64)
LnPM_10__lag1	1.240 *	1.115 **	2.058 *	−0.540	−0.893	−1.194	−1.329	−4.787 ***
	(2.07)	(2.43)	(2.07)	(−0.49)	(−0.97)	(−1.50)	(−0.94)	(−3.28)
LnNO_2__lag2	0.907	0.891 **	0.612	0.583	1.573 ***	1.452 ***	1.970 **	−1.472
	(1.68)	(2.57)	(1.02)	(0.63)	(3.54)	(3.84)	(2.70)	(−1.61)
LnSO_2__lag2	1.125 **	1.093 ***	1.824 ***	−0.356	1.993 ***	2.104 ***	2.518 ***	2.759 ***
	(3.01)	(3.94)	(4.15)	(−0.58)	(4.33)	(4.25)	(3.66)	(5.52)
LnPM_10__lag2	1.077 *	0.053	3.219 **	−2.080	1.908 *	1.135	2.842 *	4.429 ***
	(1.99)	(0.08)	(2.99)	(−1.71)	(2.08)	(1.27)	(1.85)	(4.41)
LnNO_2__lag3	0.831 *	0.850 ***	1.448 *	−0.445	2.005 **	2.081 ***	2.633 *	3.884 **
	(1.98)	(3.78)	(2.14)	(−0.60)	(2.53)	(3.75)	(1.94)	(2.73)
LnSO_2__lag3	−1.213 **	−1.245 ***	−2.201 ***	0.470	−2.283 ***	−2.271 ***	−2.726 **	−2.085 **
	(−2.97)	(−4.94)	(−3.99)	(0.57)	(−4.81)	(−4.45)	(−3.17)	(−2.52)
LnPM_10__lag3	0.342	−0.075	0.097	1.069	1.063	0.342	0.450	1.129
	(0.67)	(−0.17)	(0.13)	(1.29)	(1.28)	(0.34)	(0.34)	(1.03)
Interaction of LnSO_2_ and southern cities		−0.569 **				−0.710		
		(−2.41)				(−1.20)		
Interaction of LnNO_2_ and southern cities		2.228 *				2.259 *		
		(2.19)				(2.17)		
**R-square**	0.904	0.937	0.874	0.786	0.929	0.945	0.882	0.901
**adj. R-square**	0.777	0.84	0.708	0.491	0.822	0.84	0.702	0.74

Note: * *p* < 0.1 ** *p* < 0.05 *** *p* < 0.01. T--statistics in parentheses. #: Interaction terms added. “Ln”: Logarithm. “Δ Ln”: Log--difference. Coefficients of control variables, individual fixed--effects, and time fixed--effects were omitted for parsimony. Coefficients keep three decimals, and T--statistics keep two decimals.

**Table 3:** Cumulative health effect of air pollution on NPC incidence and Its Growth rate.

	Overall	Male	Female	Overall#	Male#	Female#
Σ LnNO_2_	3.40 ***	5.69 ***	−0.042	8.90 ***	12.26 ***	9.48 ***
	(5.62)	(5.54)	(−0.04)	(5.75)	(5.04)	(4.56)
Σ LnSO_2_	1.55	−2.03	11.12 *	6.32 *	8.30	−8.26
	(0.81)	(−0.53)	(2.13)	(1.94)	(1.41)	(−1.59)
Σ LnPM_10_	2.15 **	4.09 ***	0.39	3.54 **	3.39	2.60
	(2.26)	(3.32)	(0.26)	(2.87)	(1.25)	(1.05)
**adj. R-square**	0.78	0.71	0.49	0.82	0.70	0.74
**AIC**	−134.1	−87.07	−62.16	−110.20	−46.43	−71.88

Note: Σ means cumulative health effects of a 1% increase in air pollutants concentrations annually lasting for four years. #: NPC incidence growth rate. T--statistics in parentheses. * *p* < 0.1, ** *p* < 0.05, *** *p* < 0.01. Numbers keep two decimals.

**Table 4:** Risks (95% CIs) of NPC incidence associated with a 10 μg/m^3^ increase in ambient NO_2_, SO_2_, or PM_10_ concentrations.

	Overall	Male	Female
	One-pollutant model with zero lag (Model 1)		
NO_2_	1.1582 (0.8082 to 1.6616)	1.2774 (0.8711 to 1.8739)	1.1264 (0.9380 to 1.3530)
SO_2_	1.0827 (0.9560 to 1.2275)	1.0863 (0.8958 to 1.3185)	1.0899 (1.0125 to 1.1733) **
PM_10_	0.9841 (0.9361 to 1.0349)	0.9724 (0.8790 to 1.0742)	1.0685 (0.9930 to 1.1502) *
	One-pollutant model with three lags (Model 2)		
	NO_2_		
NO_2_	1.2193 (1.0191 to 1.4591) **	1.3189 (1.0040 to 1.7327) **	1.1883 (0.9970 to 1.4174) *
NO_2__lag1	0.7835 (0.6219 to 0.9881) *	0.8090 (0.5963 to 1.0968)	0.8521 (0.6697 to 1.0832)
NO_2__lag2	1.4019 (1.0037 to 1.9579) **	1.4902 (0.9446 to 2.3514) *	1.0711 (0.7619 to 1.5073)
NO_2__lag3	0.9627 (0.8130 to 1.1397)	0.9455 (0.7283 to 1.2275)	0.9773 (0.7718 to 1.2373)
	SO_2_		
SO_2_	1.0521 (0.9315 to 1.1890)	1.0557 (0.8530 to 1.3076)	1.1176 (1.0446 to 1.1957) ***
SO_2__lag1	1.0000 (1.0000 to 1.0000)	1.0000 (1.0000 to 1.0000)	1.0000 (1.0000 to 1.0000)
SO_2__lag2	1.0875 (0.8869 to 1.3340)	1.0893 (0.8932 to 1.3295)	0.9066 (0.7283 to 1.1281)
SO_2__lag3	0.9881 (0.8538 to 1.1434)	0.9714 (0.7611 to 1.2397)	1.0493 (0.9094 to 1.2118)
	PM_10_		
PM_10_	0.9930 (0.9352 to 1.0533)	0.9724 (0.8624 to 1.0967)	1.1109 (1.0135 to 1.2177) **
PM_10__lag1	1.0040 (0.8878 to 1.13560)	1.0178 (0.8361 to 1.2391)	0.9305 (0.8106 to 1.0682)
PM_10__lag2	0.9666 (0.8590 to 1.0874)	0.9861 (0.8187 to 1.1868)	0.9484 (0.8303 to 1.0837)
PM_10__lag3	0.9753 (0.8403 to 1.1318)	0.9637 (0.7718 to 1.2038)	0.9685 (0.9176 to 1.0227)
	Three-pollutant model with three lags (Model 3)		
	NO_2_		
NO_2_	1.5062 (1.2278 to 1.8478) ***	2.2995 (1.2567 to 4.2075) **	1.3010 (0.8212 to 2.0620)
NO_2__lag1	0.8403 (0.6914 to 1.0211) *	0.9531 (0.7305 to 1.2442)	0.8106 (0.5724 to 1.1481)
NO_2__lag2	1.2843 (1.0408 to 1.5848) **	1.1663 (0.9436 to 1.4418)	1.1062 (0.7233 to 1.6935)
NO_2__lag3	1.1589 (1.0021 to 1.3404) **	1.3767 (0.9812 to 1.9325) *	1.0925 (0.7520 to 1.5876)
	SO_2_		
SO_2_	1.0579 (0.9130 to 1.2265)	1.0993 (0.9057 to 1.3351)	0.9484 (0.7535 to 1.1926)
SO_2__lag1	1.0000 (1.0000 to 1.0000)	1.0000 (1.0000 to 1.0000)	1.0001 (1.0000 to 1.0003) **
SO_2__lag2	1.1477 (0.9570 to 1.3770)	1.3755 (0.9103 to 2.0788)	0.9560 (0.6697 to 1.3644)
SO_2__lag3	0.8949 (0.7611 to 1.0509)	0.7054 (0.4681 to 1.0624) *	1.1124 (0.7565 to 1.6362)
	PM_10_		
PM_10_	0.9560 (0.8146 to 1.1205)	0.8878 (0.7026 to 1.1224)	1.2649 (1.0674 to 1.4990) **
PM_10__lag1	1.0842 (0.9250 to 1.2714)	1.1129 (0.8454 to 1.4655)	0.9380 (0.7379 to 1.1904)
PM_10__lag2	1.0453 (0.9831 to 1.1116)	1.3252 (0.9841 to 1.7853) *	0.8361 (0.6250 to 1.1166)
PM_10__lag3	0.9743 (0.8369 to 1.1345)	0.9399 (0.7819 to 1.1289)	1.0000 (0.7804 to 1.2817)
	Cumulative relative risks (Model 4)		
∑ NO_2_	1.8836 (1.2416 to 2.8577) **	3.5187 (1.2479 to 9.9215) **	1.2742 (0.7161 to 2.2696)
∑ SO_2_	1.0857 (0.9474 to 1.2450)	1.0662 (0.8746 to 1.3002)	1.0080 (0.8212 to 1.2384)
∑ PM_10_	1.0547 (0.8790 to 1.2663)	1.2300 (0.8958 to 1.6893)	0.9900 (0.7320 to 1.3394)

Note: Σ means the relative risk of NPC incidence associated with a 10 μg/m^3^ increase in air pollutants concentrations annually lasting for four years. * *p* < 0.1, ** *p* < 0.05, *** *p* < 0.01. 95% CI in parentheses. Numbers keep four decimals.

## Appendix A

**Table A1:** Association between air pollution and NPC incidence and NPC incidence growth rate (Estimation result of control variables).

	Ln NPC Incidence				Δ ln NPC Incidence			
	*Overall*	*Overall#*	*Male*	*Female*	*Overall*	*Overall#*	*Male*	*Female*
Δ Ln income	−1.902 ***	−2.038 ***	−3.038 ***	0.763	−3.066 ***	−3.379 ***	−4.379 ***	−1.202
Ln precipitation	−0.224	−0.288	−0.5045 *	0.316	0.0195	0.0670	0.0602	1.343 **
Ln humidity	1.196 *	1.583 **	1.746	0.863	4.777 ***	5.086 ***	5.112 **	9.156 ***
Ln air facilities	−0.193 ***	−0.191 ***	−0.247 ***	−0.108	−0.511 ***	−0.539 ***	−0.525 ***	−1.009 ***
Ln private cars	−0.286	−0.0769	−0.6181 *	0.669	−0.861 ***	−0.813 ***	−0.981 *	−1.202 ***
Ln sunshine	1.084 ***	0.836 ***	1.954 ***	−0.656	2.286 ***	2.103 ***	3.267 ***	1.242 *
Ln green cover	0.104	0.804	1.243	−0.753	−0.721	−0.486	0.340	−5.097 ***
Ln hospital	−0.357 ***	−0.446 ***	−0.440 **	−0.297	−0.428 ***	−0.549 ***	−0.5488 *	−0.570 ***
Ln 2nd Industry	0.551	0.233	0.0173	1.167	−1.962 **	−2.579 **	−2.915 ***	−6.058 ***
Δ Ln smoke and alcohol	0.928 **	0.866 ***	0.958 *	−0.348	0.548	0.431	0.392	−2.042 **
Ln Edu personnel	−0.572	−0.8961 *	−1.3591 *	−0.701	−2.939 ***	−3.371 ***	−5.182 **	−4.264 ***

Note: * *p* < 0.1 ** *p* < 0.05 *** *p* < 0.01. #: Interaction terms added. “ln”: Logarithm. “Δ ln”: Log--difference. #: Models with interaction terms.

**Table A2:** Sensitivity analysis of association between air pollution and NPC incidence by sex.

	Ln NPC Incidence							
	Female					Male		
	(1)	(2)	(3)	(4)	(5)	(6)	(7)	(8)
lnNO_2_	0.75 *	1.47 **	1.18	0.599	0.942	1.03	1.96 **	4.18 ***
lnSO_2_	0.15	0.0464	0.0417	0.098	0.428	0.478	0.227	0.345
lnPM_10_	0.783	1.61 **	1.76 **	1.94 **	−0.689	−1.24 *	−1.15	−1.29 *
lnNO_2__lag1		−0.645	−0.73	−0.779		−0.202	−0.684	−0.546
lnSO_2__lag1		7.23 **	7.91 **	10.9 *		−3.27	3.19	−2
lnPM_10__lag1		−0.586	−0.618	−0.54		1.2	1.84	2.06 *
lnNO_2__lag2			0.274	0.583			1.78 **	0.612
lnSO_2__lag2			−0.0367	−0.356			0.33	1.82 ***
lnPM_10__lag2			−0.849	−2.08			0.601	3.22 **
lnNO_2__lag3				−0.445				1.45 *
lnSO_2__lag3				0.47				−2.2 ***
lnPM_10__lag3				1.07				0.0973
adj. R-square	0.496	0.555	0.519	0.491	0.319	0.297	0.544	0.708

Note: * *p* < 0.1, ** *p* < 0.05, *** *p* < 0.01.

**Table A3:** Sensitive analysis of associations between air pollution and NPC incidence growth rate by sex.

	Δ ln NPC Incidence							
	Female					Male		
	(1)	(2)	(3)	(4)	(5)	(6)	(7)	(8)
lnNO_2_	2.35 ***	2.27 **	3.99 ***	7.77 ***	3.3 ***	3.76 ***	5.21 ***	8.51 ***
lnSO_2_	0.257	0.306	0.0971	0.0835	0.383	0.236	−0.169	0.0483
lnPM_10_	1.07	1.29	0.627	1.83	−0.472	0.0415	1.45	1.42
lnNO_2__lag1		−1.28	−1.03 **	−0.698 *		−0.512	−0.956	−0.856 *
lnSO_2__lag1		−9.09	−12.4 **	−9.02		5.64	13.6	8.46
lnPM_10__lag1		−2.82	−3.6 *	−4.79 ***		0.437	−1.34	−1.33
lnNO_2__lag2			0.0989	−1.47			3.76 ***	1.97 **
lnSO_2__lag2			1.21	2.76 ***			0.895	2.52 ***
lnPM_10__lag2			2.57 ***	4.43 ***			−0.489	2.84 *
lnNO_2__lag3				3.88 **				2.63 *
lnSO_2__lag3				−2.09 **				−2.73 **
lnPM_10__lag3				1.13				0.45
adj. R-square	0.328	0.411	0.556	0.74	0.332	0.274	0.625	0.702

Note: * *p* < 0.1, ** *p* < 0.05, *** *p* < 0.01.

**Table A4:** Sensitivity analysis of relative risks (95% CI) of NPC incidence associated with a 10 μg/m^3^ increase in air pollutant concentration, adjusted only for meteorological variables with or without lagged terms.

	Three-Pollutants Model with Zero Lag	One-Pollutant Model with Zero Lag (NO_2_)	One-Pollutants Model with Three Lags (NO_2_)	One-Pollutant Model with Zero Lag (SO_2_)	One-Pollutants Model with Three Lags (SO_2_)	One-Pollutant Model with Zero Lag (PM_10_)	One-Pollutants Model with Three Lags (PM_10_)	Three-Pollutants Model with Three Lags
NO_2_	1.1026	1.122 *	1.0862 *					1.0663
SO_2_	1.0428			1.0531	1.0411			1.0854
PM_10_	0.9846					1.0103	1.0005	0.9393
NO_2_ lag1			0.8872					0.8656
SO_2_ lag1					1			1
PM_10_ lag1							1.0448	1.1129 **
NO_2_ lag2			1.1384					1.2782 **
SO_2_ lag2					1.0026			0.9279
PM_10_ lag2							0.9734	0.9254
NO_2_ lag3			1.043					1.0368
SO_2_ lag3					0.9965			1.0123
PM_10_ lag3							1.0238	1.0027

Note: * *p* < 0.1, ** *p* < 0.05.

## References

[1] Sinha S.N., Nag P.K. (2011). Air Pollution from Solid Fuels. Encycl. Environ. Health.

[2] Wichmann J., Voyi K. (2012). Ambient Air Pollution Exposure and Respiratory, Cardiovascular and Cerebrovascular Mortality in Cape Town, South Africa: 2001–2006. Int. J. Environ. Res. Public Health.

[3] Cohen A.J., Brauer M., Burnett R., Anderson H.R., Frostad J., Estep K., Balakrishnan K., Brunekreef B., Dandona L., Dandona R. (2017). Estimates and 25-year trends of the global burden of disease attributable to ambient air pollution: An analysis of data from the Global Burden of Diseases Study 2015. Lancet.

[4] Key Facts of Ambient (outdoor) Air Pollution. World Health Organization. https://www.who.int/en/news-room/fact-sheets/detail/ambient-(outdoor)-air-quality-and-health.

[5] Gulisano M., Marceddu S., Barbaro A., Pacini A., Buiatti E., Martini A., Pacini P. (1997). Damage to the nasopharyngeal mucosa induced by current levels of urban air pollution: A field study in lambs. Eur. Respir. J..

[6] Ho C.K., Lo W.C., Huang P.H., Wu M.T., Christiani D.C., Lin C.T. (1999). Suspected nasopharyngeal carcinoma in three workers with long-term exposure to sulphuric acid vapour. Occup. Environ. Med..

[7] Xu X., Ding H., Wang X. (1995). Acute effects of total suspended particles and sulfur dioxides on preterm delivery: A community-based cohort study. Arch. Environ. Health.

[8] Donaldson K., Tran L., Jimenez L.A., Duffin R., Newby D.E., Mills N., MacNee W., Stone V. (2005). Combustion-derived nanoparticles: A review of their toxicology following inhalation exposure. Part. Fibre Toxicol..

[9] Cormier S.A., Lomnicki S., Backes W., Dellinger B. (2006). Origin and health impacts of emissions of toxic by-products and fine particles from combustion and thermal treatment of hazardous wastes and materials. Environ. Health Perspect..

[10] Pichichero M.E., Almudevar A. (2016). Inflammation-associated cytokine analysis identifies presence of respiratory bacterial pathogens in the nasopharynx. Pathog. Dis..

[11] Hamra G.B., Laden F., Cohen A.J., Raaschou-Nielsen O., Brauer M., Loomis D. (2015). Lung Cancer and Exposure to Nitrogen Dioxide and Traffic: A Systematic Review and Meta-Analysis. Environ. Health Perspect..

[12] Bourouba M., Zergoun A.A., Maffei J.S., Chila D., Djennaoui D., Asselah F., Amir-Tidadini Z.C., Touil-Boukoffa C., Zaman M.H. (2015). TNFα antagonization alters NOS_2_ dependent nasopharyngeal carcinoma tumor growth. Cytokine.

[13] Postlethwait E.M., Bidani A. (1989). Pulmonary disposition of inhaled NO2-nitrogen in isolated rat lungs. Toxicol. Appl. Pharmacol..

[14] Ward M.H., Pan W.H., Cheng Y.J., Li F.H., Brinton L.A., Chen C.J., Hsu M.M., Chen I.H., Levine P.H., Yang C.S. (2000). Dietary exposure to nitrite and nitrosamines and risk of nasopharyngeal carcinoma in Taiwan. Int. J. Cancer.

[15] Fan H.C., Chen C.Y., Hsu Y.C., Chou R.H., Teng C.J., Chiu C.H., Hsu C.Y., Muo C.H., Chang M.Y., Chang K.H. (2018). Increased risk of incident nasopharyngeal carcinoma with exposure to air pollution. PLoS ONE.

[16] Beelen R., Raaschou-Nielsen O., Stafoggia M., Andersen Z.J., Weinmayr G., Hoffmann B., Wolf K., Samoli E., Fischer P., Nieuwenhuijsen M. (2014). Effects of long-term exposure to air pollution on natural-cause mortality: An analysis of 22 European cohorts within the multicentre ESCAPE project. Lancet.

[17] Zhou M., He G., Liu Y., Yin P., Li Y., Kan H., Fan M., Xue A., Fan M. (2015). The associations between ambient air pollution and adult respiratory mortality in 32 major Chinese cities, 2006–2010. Environ. Res..

[18] Aliyu A.J., Ismail N.W. (2016). The effects of air pollution on human mortality: Does gender difference matter in African countries?. Environ. Sci. Pollut. Res. Int..

[19] Lv J.W., Huang X.D., Chen Y.P., Zhou G.Q., Tang L.L., Mao Y.P., Li W.F., Lin A.H., Ma J., Sun Y. (2018). A National Study of Survival Trends and Conditional Survival in Nasopharyngeal Carcinoma: Analysis of the National Population-Based Surveillance Epidemiology and End Results Registry. Cancer Res. Treat..

[20] Elliott P., Shaddick G., Wakefield J.C., de Hoogh C., Briggs D.J. (2007). Long-term associations of outdoor air pollution with mortality in Great Britain. Thorax.

[21] A Notice on the Issuance of China’s Three-year Action Plan on Cancer Prevention and Control (2015–2017). Chinese Ministry of Health. http://www.nhc.gov.cn/jkj/s5878/201509/656437bc5c7e4cd0afb581de85be998a.shtml.

[22] 2018 Cancer Fact Sheets of Nasopharynx. World Health Organization. https://gco.iarc.fr/today/data/factsheets/cancers/4-Nasopharynx-fact-sheet.pdf.

[23] 2018 Population Fact Sheets of China. World Health Organization. https://gco.iarc.fr/today/data/factsheets/populations/160-china-fact-sheets.pdf.

[24] Jia J., Cheng S., Lei L., Lang J. (2017). An Iegrated WRFx-CAMx Modeling Approach for Impact Analysis of Implementing the Emergency PM_2.5_ Control Measures during Red Alerts in Beijing in December 2015. Aerosol Air Qual. Res..

[25] Kanaya Y., Pan X.L., Miyakawa T., Komazaki Y., Taketani F., Uno I., Kondo Y. (2016). Long-term observations of black carbon mass concentrations at Fukue Island, western Japan, during 2009–2015: Constraining wet removal rates and emission strengths from East Asia. Atmos. Chem. Phys..

[26] Chuang M.T., Chou C.C.K., Lin N.H., Takami A., Hsiao T.C., Lin T.H., Fu J.S., Pani S.K., Lu Y.R., Yang T.Y. (2017). A Simulation Study on PM_2.5_ Sources and Meteorological Characteristics at the Northern Tip of Taiwan in the Early Stage of the Asian Haze Period. Aerosol Air Qual. Res..

[27] Sahu L.K., Kondo Y., Miyazaki Y., Kuwata M., Koike M., Takegawa N., Tanimoto H., Matsueda H., Yoon S.C., Kim Y.J. (2009). Anthropogenic aerosols observed in Asian continental outflow at Jeju Island, Korea, in spring 2005. J. Geophys. Res..

[28] Zhu C., Kawamura K., Kunwar B. (2015). Effect of biomass burning over the western North Pacific Rim: Wintertime maxima of anhydrosugars in ambient aerosols from Okinawa. Atmos. Chem. Phys..

[29] Gao R., Wang L., Ye Y.F., Du J.L., Chen S.H., Guo J., Yang M.J., Lin C.Y., Lin Q., Cao S.M. (2017). Evaluation of seven recombinant VCA-IgA ELISA kits for the diagnosis of nasopharyngeal carcinoma in China: A case-control trial. BMJ Open.

[30] Zhao J.J., Shi X.C., Wang K.L., Yu W.H., Yin H.C. (2017). The Influence of Land Intensive Use and Urbanization to Air Pollution: Evidence from China. IOP Conf. Ser. Earth Environ. Sci..

[31] Huang H.B., Lai C.H., Chen G.W., Lin Y.Y., Jaakkola J.J., Liou S.H., Wang S.L. (2012). Traffic-related air pollution and DNA damage: A longitudinal study in Taiwanese traffic conductors. PLoS ONE.

[32] Zheng Y.M., Tuppin P., Hubert A., Jeannel D., Pan Y.J., Zeng Y., de Thé G. (1994). Environmental and dietary risk factors for nasopharyngeal carcinoma: A case-control study in Zangwu County, Guangxi, China. Br. J. Cancer.

[33] Beeson L., Abbey D.E., Knutsen S. (1998). Long-term ambient concentrations of selected air pollutants and incident malignant neoplasms in california adults: Results from the AHSMOG study. Epidemiology.

[34] Giovanis E. (2015). Relationship between recycling rate and air pollution: Waste management in the state of Massachusetts. Waste Manag..

[35] Heinrich J., Thiering E., Rzehak P., Krämer U., Hochadel M., Rauchfuss K.M., Gehring U., Wichmann H.E. (2013). Long-term exposure to NO_2_ and PM_10_ and all-cause and cause-specific mortality in a prospective cohort of women. Occup. Environ. Med..

[36] Liu W.L., Xu Z.P., Yang T.A. (2018). Health effects of air pollution in China. Int. J. Environ. Res. Public Health.

[37] Qiu X., Duan L., Cai S., Yu Q., Wang S., Chai F., Gao J., Li Y., Xu Z. (2017). Effect of current emission abatement strategies on air quality improvement in China: A case study of Baotou, a typical industrial city in Inner Mongolia. J. Environ. Sci..

[38] Samoli E., Schwartz J., Wojtyniak B., Touloumi G., Spix C., Balducci F., Medina S., Rossi G., Sunyer J., Bacharova L. (2001). Investigating regional differences in short-term effects of air pollution on daily mortality in the APHEA project: A sensitivity analysis for controlling long-term trends and seasonality. Environ. Health Perspect..

[39] Bernanke B., James H. (1990). The Gold Standard, Deflation, and Financial Crisis in the Great Depression: An International Comparison. NBER Work. Paper.

[40] Aiken L.S., West S.G. (1991). Multiple Regression: Testing and Interpreting Interactions.

[41] Whittington L.A., Alm J., Peters H.E. (1990). Fertility and the Personal Exemption: Implicit Pronatalist Policy in the United States. Am. Econ. Rev..

[42] Zhang F., Li L., Krafft T., Lv J., Wang W., Pei D. (2011). Study on the association between ambient air pollution and daily cardiovascular and respiratory mortality in an urban district of Beijing. Int. J. Environ. Res. Public Health.

[43] Air Quality Guidelines. Global update 2005. Particulate Matter, Ozone, Nitrogen Dioxide and Sulfur Dioxide. World Health Organization. http://www.euro.who.int/__data/assets/pdf_file/0005/78638/E90038.pdf?ua=1.

[44] Aunan K., Pan X.C. (2004). Exposure-response functions for health effects of ambient air pollution applicable for China—A meta-analysis. Sci. Total Environ..

[45] Xie S.H., Yu I.T., Tse L.A., Au J.S., Wang F., Lau J.S., Zhang B. (2014). Domestic incense burning and nasopharyngeal carcinoma: A case-control study in Hong Kong Chinese. Environ. Mol. Mutagen..

[46] He Y.Q., Xue W.Q., Shen G.P., Tang L.L., Zeng Y.X., Jia W.H. (2015). Household inhalants exposure and nasopharyngeal carcinoma risk: A large-scale case-control study in Guangdong, China. BMC Cancer.

[47] Chen Y.P., Chan A.T.C., Le Q.T., Blanchard P., Sun Y., Ma J. (2019). Nasopharyngeal carcinoma. Lancet.

[48] Paul P., Deka H., Malakar A.K., Halder B., Chakraborty S. (2018). Nasopharyngeal carcinoma: Understanding its molecular biology at a fine scale. Eur. J. Cancer Prev..

[49] Yuan J.M., Wang X.L., Xiang Y.B., Gao Y.T., Ross R.K., Yu M.C. (2000). Non-dietary risk factors for nasopharyngeal carcinoma in Shanghai, China. Int. J. Cancer.

[50] Verma V., Fang T., Guo H.Y., King L.E., Bates J.T., Peltier R.E., Edgerton E., Russell A.J., Weber R.J. (2014). Reactive oxygen species associated with water-soluble PM_2.5_ in the southeastern United States: Spatiotemporal trends and source apportionment. Atmos. Chem. Phys..

[51] Lammel G., Cape J.N. (1996). Nitrous acid and nitrite in the atmosphere. Chem. Soc. Rev..

[52] Nitrogen Dioxide Pollution Mapped. European Space Agency. http://www.esa.int/Applications/Observing_the_Earth/Copernicus/Sentinel-5P/Nitrogen_dioxide_pollution_mapped.

[53] Sun H., Geng Y., Hu L., Shi L., Xu T. (2018). Measuring China’s new energy vehicle patents: A social network analysis approach. Energy.

[54] Sun H., Bless K.E., Sun C., Kporsu A.K. (2019). Institutional quality, green innovation and energy efficiency. Energy Policy.

